# Development of a Prediction Method of Cell Density in Autotrophic/Heterotrophic Microorganism Mixtures by Machine Learning Using Absorbance Spectrum Data

**DOI:** 10.3390/biotech11040046

**Published:** 2022-10-12

**Authors:** Akihito Nakanishi, Hiroaki Fukunishi, Riri Matsumoto, Fumihito Eguchi

**Affiliations:** 1 School of Bioscience and Biotechnology, Tokyo University of Technology, Hachioji 192-0982, Japan; 2 Graduate School of Bionics, Tokyo University of Technology, Hachioji 192-0982, Japan; 3 School of Computer Sciences, Tokyo University of Technology, Hachioji 192-0982, Japan; 4 Graduate School of Computer Sciences, Tokyo University of Technology, Hachioji 192-0982, Japan

**Keywords:** microflora, explainable artificial intelligence (XAI), extremely randomized trees regressor, Shapley additive explanations (SHAPs)

## Abstract

Microflora is actively used to produce value-added materials in industry, and each cell density should be controlled for stable microflora use. In this study, a simple system evaluating the cell density was constructed with artificial intelligence (AI) using the absorbance spectra data of microflora. To set up the system, the prediction system for cell density based on machine learning was constructed using the spectra data as the feature from the mixture of *Saccharomyces cerevisiae* and *Chlamydomonas reinhardtii*. As the results of predicting cell density by extremely randomized trees, when the cell densities of *S. cerevisiae* and *C. reinhardtii* were shifted and fixed, the coefficient of determination (*R*^2^) was 0.8495; on the other hand, when the cell densities of *S. cerevisiae* and *C. reinhardtii* were fixed and shifted, the *R*^2^ was 0.9232. To explain the prediction system, the randomized trees regressor of the decision tree-based ensemble learning method as the machine learning algorithm and Shapley additive explanations (SHAPs) as the explainable AI (XAI) to interpret the features contributing to the prediction results were used. As a result of the SHAP analyses, not only the optical density, but also the absorbance of the Soret and Q bands derived from the chloroplasts of C. reinhardtii could contribute to the prediction as the features. The simple cell density evaluating system could have an industrial impact.

## 1. Introduction

The production of value-added materials such as alcohol and amino acids has been performed by using microorganisms for a long time [1,2]. Improving the material productivity by these microorganisms is a major industrial challenge [3], and the productivity improvement has been attempted based on knowledge and experiences regarding the culturing conditions such as the microbial strains and media [4]. However, the improvements derived from the knowledge and experiences are limited and have been attempted with comprehensive analyses of the culture conditions [5]. Recently, regarding the microorganisms’ condition in the culture, comprehensive analyses focusing on omics such as metabolomics and transcriptomics have been performed with artificial intelligence (AI) [6]. AI technology, more specifically machine learning, recently has been applied to the analysis tools in microbiology research for microbial classification and the interaction between microorganisms and the surrounding environment [7,8]. By using machine learning models that have learned patterns in known data, tedious, time-consuming, and error-prone tasks can be performed more quickly and accurately. Therefore, this research proposes a cell density prediction method using absorbance spectrum information as a large amount of data and machine learning for the advancement of biotechnology. Deep-learning- [9] or tree-based ensemble learning methods, such as XGBoost [10], LightGBM [11], and CatBoost [12] as the boosting methods, and random forest [13] and extremely randomized trees [14] as the bagging methods have been widely used as the high-performance machine learning algorithms. Deep learning has strengths with respect to unstructured data such as images, audio, and text data, while the tree-based ensemble learning methods have strengths with respect to table-style numerical data. In this study, an ensemble learning method, specifically extremely randomized trees, was used because the input data were table-style numerical data. In recent years, machine learning has achieved significant performance gains due to the increasing complexity of models. With increasing complexity, these models have become black boxes, introducing uncertainty about the final outcome. Deep-learning- [9] or tree-based ensemble learning methods [10,11,12,13,14] have been classified as black box models. The awareness of this issue has led to a great deal of scientific interest in the field of explainable artificial intelligence (XAI) [15], which is studied to develop methods to explain and interpret machine learning models. Various XAI methods are available for different types of machine learning models and different types of data formats, but the technology is still in the developmental stages. Among these, Shapley additive explanations (SHAPs) [16] comprise a more complete method, which can be applied to any machine learning model and any type of data and be useful to interpret the contributions of the features to the prediction results at both the global and local level. SHAPs are based on the game theoretical optimal Shapley values [17], which represent the contribution of each player in a multi-player game when players cooperate. In SHAPs, the multiple players are equivalent to the features used in the machine learning model, and the contributions of the features to individual predictions are calculated to enhance the interpretability. So far, although omics analyses with AI have been performed on the cultures of single microorganisms such as *Escherichia coli* [18] and *Saccharomyces cerevisiae* [19], the analyses of more complicated microbial flora including meta-omics have not been sufficient. Therefore, the XAI method has been required to understand the microbial condition from the viewpoint of meta-omics. Additionally, in the field of microbiology, there are few reports of research revealing microbial phenomena using AI [20,21]; therefore, our study has an impact on this field.

**Figure 1:**
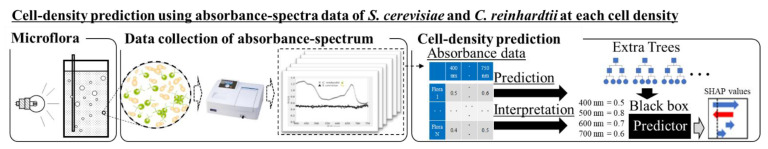
The concept of cell density prediction in microbial flora using absorbance spectra data.

In this study, the research purpose was to exhibit a method easily predicting the cell density in the mixture of *C. reinhardtii* and *S. cerevisiae* using machine learning with the absorbance spectrum, to explain the reason why the method to predict the cell density of the mixture works with the absorbance spectrum as XAI using SHAPs. To develop the cell density prediction method, (i) the Extra Trees algorithm to predict the cell density using the absorbance spectra data and (ii) the SHAP algorithm to evaluate the Extra Trees algorithm were set up (Figure 1). The cell-containing solution was composed of a photosynthetic microorganism, which provides substrates derived from CO_2_ as the carbon source, and a heterotrophic microorganism, which can use the substrates. This combination is important from the viewpoint of industry because even heterotrophic microorganisms can produce useful substances directly from carbon dioxide through the organification of carbon dioxide by autotrophic microorganisms. Firstly, absorbance spectra in various cell densities of *Chlamydomonas reinhardtii* and *S. cerevisiae* were obtained to set up the cell density prediction method as essential data for machine learning. Secondly, machine learning was performed using the data of the obtained cell density and absorbance spectra to clarify each relationship. In this study, one of the challenges was to explain which absorbance wavelengths affect cell density with respect to the machine learning model, in addition to evaluating the performance of the cell density prediction. There is no report about predicting the cell density of autotrophic/heterotrophic microorganism mixtures by machine learning using absorbance spectra data; therefore, this research can show the impact for industry.

## 2. Materials and Methods

### 2.1. Strains and Growth Conditions

The *S. cerevisiae* BY4741 strain (*MAT*a, *his3Δ1*, *leu2Δ0*, *met15Δ0*, *ura3Δ0*) was purchased from Funakoshi (Tokyo, Japan) as a laboratory haploid strain [22]. *S. cerevisiae* was cultured at 30 °C in YPD (yeast extract, 10 g·L^−1^; peptone, 20 g·L^−1^; glucose, 20 g·L^−1^). The *C. reinhardtii* strain C-9: NIES-2235 was provided by the National Institute for Environmental Studies. *C. reinhardtii* was cultivated under a white fluorescent light intensity of 150 μmol photons·m^−2^·s^−1^ at 25 °C with CO_2_ bubbling (15,000 ppm, 0.05 vvm) in BG˗11 [23]. All processes for *C. reinhardtii* cultivation were provided by CMD Corporation.

### 2.2. Cell Density Evaluation

For the absorbance spectrum analysis, the cell-containing solutions were composed of (i) only *S. cerevisiae*, (ii) only *C. reinhardtii*, and (iii) *S. cerevisiae* and *C. reinhardtii*, respectively. Each cell density was evaluated by absorbance from 400 nm to 750 nm in 1 nm increments with the 96-well microplate reader SH-1300Lab CRN-2508 (Hitachi High-Tech Science Corporation, Tokyo, Japan).

### 2.3. Cell Density Prediction by AI Analytics

**Figure 2:**
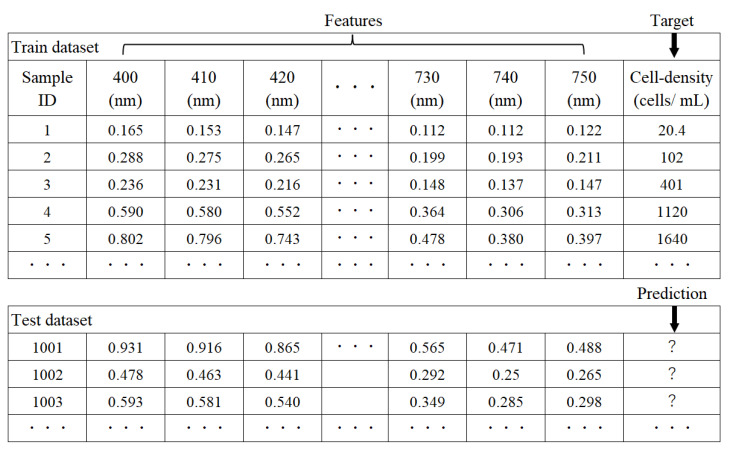
Absorbance wavelengths as features were used for cell density prediction.

**Figure 3:**
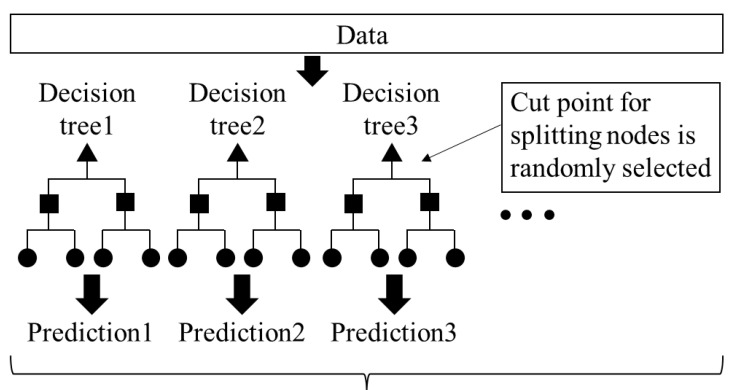
Machine learning algorithm to predict cell density with values at each absorbance wavelength as the feature to be explained (concept of the algorithm Extra Trees Regressor).

The AI analysis consisted of the two components of cell density prediction and the explanation of the prediction results. Cell density predictions using absorbance spectra data were performed for three solvent conditions containing different microorganisms: single *S. cerevisiae*, single *C. reinhardtii*, and mixture of *S. cerevisiae* and *C. reinhardtii*. The predicted target variable was the cell density for the microorganism in each solvent condition. In the third mixture solution, each cell density of *S. cerevisiae* and *C. reinhardti* was predicted using the same absorbance spectra data. To generate a prediction model, 36 features of absorbance identified at 10 increments were extracted from 400 to 750. The data structure for cell density prediction is shown in Figure 2. As the machine learning algorithm for cell density prediction, the extremely randomized trees regressor [14], also called Extra Trees, was used from the Pycaret library [24] of the Python language. Extra Trees is an ensemble learning algorithm using a set of multiple decision trees, shown as Figure 3. The main difference from other tree-based ensemble learning algorithms is that each decision tree is grown using whole training samples, but not bootstrap samples, and each node in a decision tree is split by choosing a cut-point at random, but not by calculating the locally optimal combination of the feature and cut-point based on an information criterion. The cut-point t is usually represented as $x\left[i\right]>t$, where $x\left[i\right]$ indicates the i−th feature. Final predictions in the case of regression are yielded by averaging the predictions of the multiple decision trees. By using multiple decision trees generated by random cutoffs in this way, the overfitting that occurs in a single decision tree algorithm can be improved. This corresponds to reducing the variance of the bias variance problem [14]. The hyper-parameters in Extra Trees were turned by the random search method [25] of Pycaret under the setting of 10-fold cross-validation, the generation of 100 decision trees as the predictors, and the optimization index of the coefficient of determination (*R*^2^) for the scatter plot of actual vs. predicted values. The accuracy of the cell density predictions by AI analytics based on the spectra data was assessed with accuracy ratios calculated by dividing the predicted cell density by the actual cell density.

### 2.4. Explanation of the Prediction Results

One of the challenges in this study was to elucidate which absorbance wavelengths affect cell density prediction; however, machine learning models are generally black boxes, and it is difficult to explain why the prediction values are output. Recently, however, Shapley additive explanation (SHAP) [16] analysis has received attention in the field of explainable AI (XAI) [15] to interpret how each feature affects the prediction values. The advantage of SHAPs is that the prediction values can be expressed as the sum of the effects of features and the base value estimated by the expected prediction values, i.e., the mean of all prediction values for the input samples, called additive feature attribution methods [16].

(1) $$f\left(x\right)=\phi_{0}\left(f;x\right)+\sum_{i=1}^{M}\phi_{i}\left(f;x\right)$$

where x, $f\left(x\right)$, $\phi_{0}\left(f;x\right)$, $\phi_{i}\left(f;x\right)$ M are represented as the data of the sample, the prediction value generated by the trained model f, the base value calculated as the mean of the prediction values, the SHAP value for the i-th feature, and the number of features, respectively. Therefore, it is possible to intuitively interpret the contribution of the features by how much each feature raises or lowers the predicted value from the base value. The SHAP analysis was performed by the SHAP method for tree-based machine learning models, called TreeSHAP [26] in the Python language shap library.

## 3. Results and Discussion

### 3.1. Evaluation of Absorbance Spectrum of Each Strain of S. cerevisiae and C. reinhardtii

**Figure 4:**
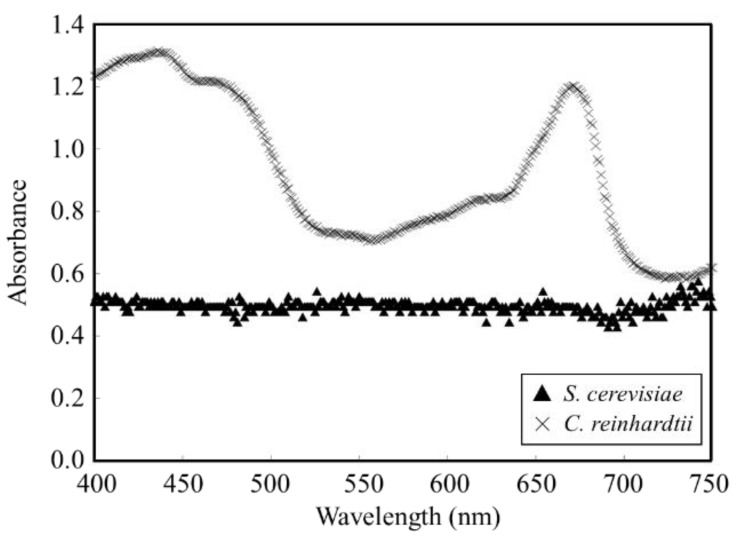
Absorbance spectra of cell-containing solution of *S. cerevisiae* as ▲ and *C. reinhardtii* as × were measured. Spectrum range was 400~750 nm (absorbance spectra of *S. cerevisiae* and *C. reinhardtii*).

The development of a method to predict cell density in a cell-containing solution composed of *S. cerevisiae* and *C. reinhardtii* was attempted with the absorbance spectrum of the microbial flora. Since the differences in the absorbance spectrum characteristics of *S. cerevisiae* and *C. reinhardtii* could be a stepping stone for predicting each cell density in the cell-containing solution, the spectra of the cell-containing solution composed of single strains of *S. cerevisiae* and *C. reinhardtii* were analyzed (Figure 4). As the results, no significant shift was observed in the absorbance spectrum of *S. cerevisiae*, and specific absorbance bands were confirmed at 425 nm and 675 nm for *C. reinhardtii*.

The cell densities of *S. cerevisiae* and *C. reinhardtii* were kept constant, and those absorbance spectra were analyzed from 400 to 750 nm. *C. reinhardtii*, a green alga, has chloroplasts for photosynthesis, and chloroplasts use light mainly in the Soret and Q bands, which are highly efficient for photosynthesis [27,28]. Therefore, *C. reinhardtii* in this study showed maximum absorbance at 425 nm and 675 nm. Since *S. cerevisiae* has no chloroplasts, the broad shape of the absorbance spectrum resulted from simple light scattering. Thus, the differences in the absorbance spectrum due to the characteristics of each cell were confirmed. The absorbance spectra were obtained from the cell-containing solution adjusted for each cell density of *S. cerevisiae* and *C. reinhardtii*, and the cell density prediction using the absorbance spectra data was attempted simply to estimate the cell density.

### 3.2. Evaluation of Predictive Performance for Cell Density Prediction with the Coefficient of Determination

**Figure 5:**
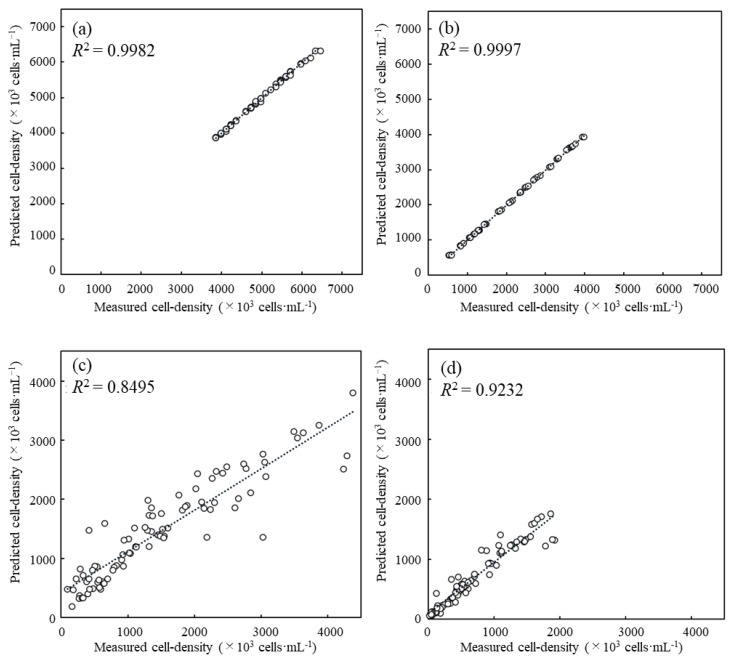
Cell density in each cell-containing solution was estimated with the prediction method using spectra data. The predicted cell density was compared with the measured value to analyze the prediction accuracy. (**a**) Only *S. cerevisiae*, drifted: *S. cerevisiae* cell density; (**b**) only *C. reinhardtii*, drifted *C. reinhardtii* cell density; (**c**) *S. cerevisiae* and *C. reinhardtii*, fixed: *C. reinhardtii* cell density, drifted: *S. cerevisiae* cell density; (**d**) *S. cerevisiae* and *C. reinhardtii*, fixed: *S. cerevisiae* cell density, drifted: *C. reinhardtii* cell density. (Evaluation for prediction accuracy of cell density in cell-containing solution using absorbance spectra.)

The cell density prediction in the cell-containing solution was made using the absorbance spectra data from the microbial flora. The scatter plots with the measured and predicted values of the cell density in the cell-containing solution were drawn to evaluate the predictive performance (Figure 5). In the scatter plots, the coefficient of determination *R*^2^ for *S. cerevisiae* and *C. reinhardtii*, which consisted of only a single strain, was 0.9982 and 0.9997, respectively, indicating that the fitted curve showed extremely high-precision linearity. The *R*^2^ was 0.8495 when the cell density was fixed in *C. reinhardtii* and shifted in *S. cerevisiae*; on the other hand, the *R*^2^ was 0.9232 when fixed in *C. reinhardtii* and shifted in *S. cerevisiae*.

According to the results of the scatter diagrams of the measured and predicted cell density, the *R*^2^ of the cell-containing solutions composed of single strains of *S. cerevisiae* and *C. reinhardtii* were extremely high, indicating that the cell density could be evaluated with high accuracy depending on the absorbance spectrum from the low- to high-density band. These are natural results because both cell densities were generally measured by optical density; however, the validity of the prediction is shown by the fact that the cell density can be accurately estimated from the absorbance spectrum of the cell-containing solutions composed of single strains. On the other hand, even the *R*^2^ of the cell-containing solution with *S. cerevisiae* and *C. reinhardtii* exceeded 0.84, indicating that the prediction could be workable. In order to understand the reason for the high *R*^2^, the impacts of absorbance at each wavelength were evaluated by using SHAP values.

### 3.3. Feature Importance Based on SHAP Values

**Figure 6:**
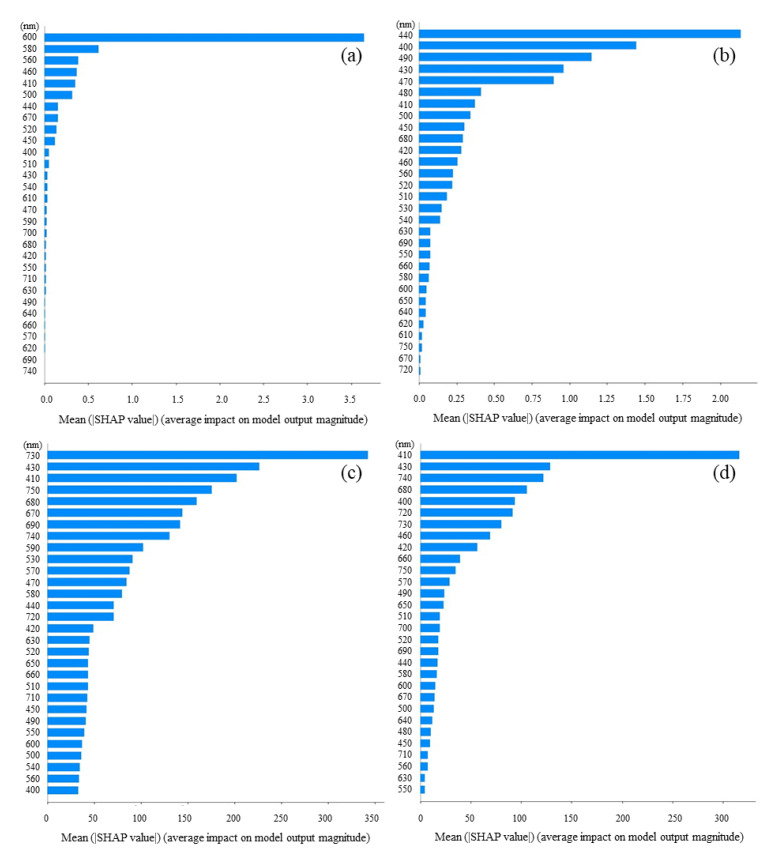
The mean absolute SHAP values for interpreting the impact of the features on the prediction results. Features are sorted in descending order of impact. (**a**) Only *S. cerevisiae*, drifted: *S. cerevisiae* cell density; (**b**) only *C. reinhardtii*, drifted *C. reinhardtii* cell density; (**c**) *S. cerevisiae* and *C. reinhardtii*, fixed: *C. reinhardtii* cell density, drifted: *S. cerevisiae* cell density; (**d**) *S. cerevisiae* and *C. reinhardtii*, fixed: *S. cerevisiae* cell density, drifted: *C. reinhardtii* cell density. (Comparison of feature importance at each wavelength as a feature analyzed by SHAP).

SHAP analyses were performed to understand how each feature value affected the predicted value of the cell density. In order to evaluate the feature importance, the mean absolute SHAP values were compared, respectively (Figure 6). In the case of the single strain of *S. cerevisiae*, the impact indicated a large value exceeding 3.5 at 600 nm, and these were less than 1.0 at the other absorbance wavelengths. On the other hand, in the case of the single strain of *C. reinhardtii*, the impacts indicated remarkably large values exceeding 2.0 at 440 nm, over 1.0 at 400 nm and 490 nm, and less than 1.0 at other wavelengths. In the case of the mixed strain of *S. cerevisiae* and *C. reinhardtii*, when the cell density of *C. reinhardtii* was fixed and the cell density of *S. cerevisiae* was shifted, the impact was significantly higher at 730 nm, exceeding 300, followed by higher values at 430 nm and 410 nm. On the other hand, when the cell density of *S. cerevisiae* was fixed and the cell density of *C. reinhardtii* was shifted, the impact was characteristically over 300 at 410 nm, followed by high values at 430 nm and 740 nm.

To show how the absorbance at each wavelength affected the cell density prediction, the mean absolute SHAP values were evaluated at wavelengths as features. In the case of *S. cerevisiae*, the impact at 600 nm was the highest in whole absorbance wavelength, and this fact corresponds to the evaluation method of the cell density of *S. cerevisiae*, generally with the OD_600_. The result indicated that the prediction of the cell density of *S. cerevisiae* with the OD_600_ was significantly reasonable. For *C. reinhardtii*, the high impacts of *C. reinhardtii* were shown at 440 nm, 400 nm, and 490 nm and coincided with the Soret absorbance band. Therefore, these facts present that the cell density prediction using absorbance spectra could be important with respect to the biological properties of *C. reinhardtii*. From the above results, the prediction was successful based on the properties of the absorbance of both strains *S. cerevisiae* and *C. reinhardtii*. Thus, even for the mixture of *S. cerevisiae* and *C. reinhardtii*, the possibility was shown that the prediction could evaluate the density based on the specific properties of the absorbance spectrum of both strains. In fact, both *R*^2^ were over 0.84 when the cell densities in the mixtures of *S. cerevisiae* and *C. reinhardtii* were evaluated with the prediction (Figure 5). When the density of *S. cerevisiae* was shifted and the one of *C. reinhardtii* was fixed, even without using the factor of absorbance at 600 nm, the highest impact for the others was shown at 730 nm, the wavelength of which is not related to the Q band derived from the chloroplasts. The results could mean that the impact was shown as the optical density relating simply to the cell density in the mixture. Additionally, high impacts were detected at 430 nm and 410 nm, and those impacts could contribute to the algorithm predicting the cell density using the spectra data because those wavelengths are in the Soret band derived from *C. reinhardtii* properties. On the other hand, when the cell density of *S. cerevisiae* was fixed and the one of *C. reinhardtii* was shifted, the results indicated that the high impacts at 470 nm and 430 nm and the one at 740 nm were related to the Soret band and the optical density, respectively. In either example, those analyses revealed which wavelengths worked effectively as the features for the prediction of the cell density using the absorbance spectra data. However, in order to understand the prediction in detail, the analyses of the positive or negative impacts on the predicted value should be required because the impacts were shown with the absolute value.

### 3.4. Contribution of Features for Each Sample Based on SHAP Values

**Figure 7:**
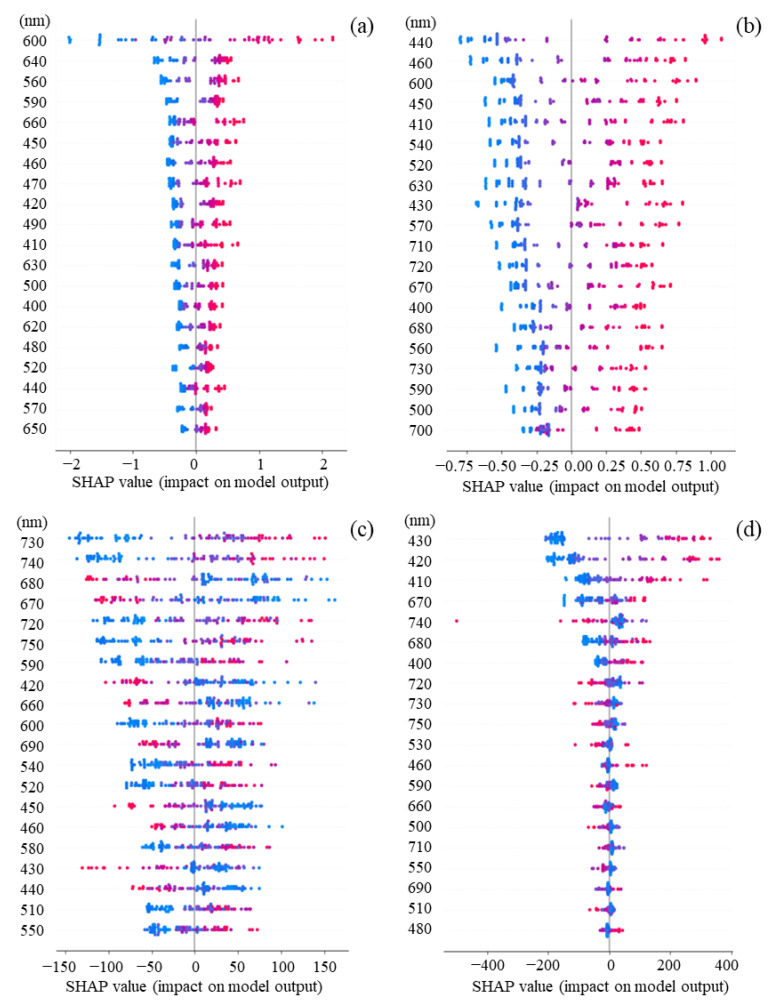
Features and impacts are the absorbance wavelengths (nm) and SHAP values, respectively. Dot diagram of SHAP values for each sample to describe the impact of the features. In the graph, features are arranged in order of the mean absolute value of the SHAP values. The horizontal axis represents the SHAP value, and the larger the SHAP value in the positive or negative direction, the more the feature contributes in each direction. The color represents the magnitude of the feature, with larger features being red and smaller features being blue. (**a**) Only *S. cerevisiae*, drifted: *S. cerevisiae* cell density; (**b**) only *C. reinhardtii*, drifted *C. reinhardtii* cell density; (**c**) *S. cerevisiae* and *C. reinhardtii*, fixed: *C. reinhardtii* cell density, drifted: *S. cerevisiae* cell density; (**d**) *S. cerevisiae* and *C. reinhardtii*, fixed: *S. cerevisiae* cell density, drifted: *C. reinhardtii* cell density. (Comparison of positive/negative impacts of absorbance at each wavelength analyzed by SHAP.) Analyses for the positive and negative impacts of each feature on cell density were performed. The reason why the values of the features and the impacts on the features were positively correlated with each other in the cell-containing solutions consisting of only a single strain could be that the spectrum properties of the observed strain was not disturbed by the properties of another strain. In fact, *S. cerevisiae* and *C. reinhardtii* are strains having different properties, and the impacts of the features of 420–450 nm and 660–690 nm had an effect in the negative direction when the density of *C. reinhardtii* was fixed and the density of *S. cerevisiae* was increased. The reason could be that the ratio of the cell density occupied by *C. reinhardtii* relatively reduced with respect to the one by *S. cerevisiae* in the cell-containing solution since the wavelengths of 420–450 nm and 660–690 nm correspond to the Soret and Q bands derived from the chloroplasts of *C. reinhardtii*. Furthermore, when the cell density of *S. cerevisiae* was fixed and the one of *C. reinhardtii* was increased, this conjecture could be also supported by the fact that the impacts were high in the negative direction at 720–750 nm. Additionally, the impacts were high in the positive direction at 400–430 nm, 460 nm, and 670–680 nm, indicating that the cell density ratio of the green alga was significantly correlated with the Soret band and Q band. Therefore, the differences in the impacts depending on the specific features of both strains could be significant for the construction of the algorithm to calculate the cell density in the cell-containing solution.

Detailed SHAP analyses were attempted by dividing the impact into positive and negative SHAP values (Figure 7). The impacts were positively correlated with the values for all features. In detail, *S. cerevisiae* and *C. reinhardtii* had positive impacts at 600 nm and around 400–500 nm, respectively. When the cell density of *C. reinhardtii* was fixed and the one of *S. cerevisiae* was shifted, the impacts were positive at 730 nm and 740 nm, but negative at 420–450 nm and 660–690 nm. On the other hand, when the cell density of *S. cerevisiae* was fixed and the one of *C. reinhardtii* was shifted, the impacts were positive at 400–430 nm, 460 nm, and 670–680 nm, but negative at 720–750 nm.

### 3.5. Evaluation of Prediction Accuracy on Each Cell Density

**Figure 8:**
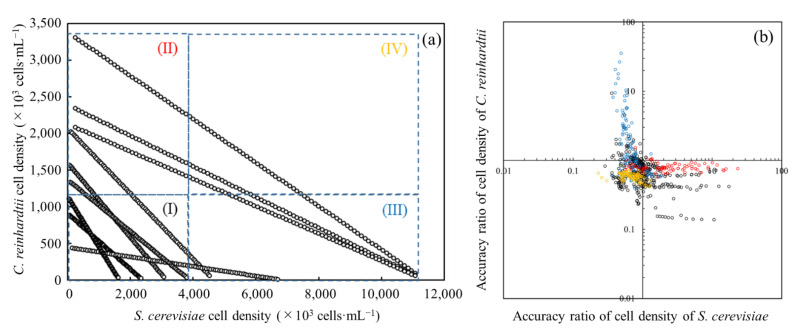
The accuracy of each cell density estimated by the prediction method using absorbance spectra data analyzed with a distribution diagram. (**a**) Each cell density in the cell-containing solution is plotted in four compartments depending on the value of each cell density as follows: (I) *S. cerevisiae*: low density, *C. reinhardtii*: low density; (II) *S. cerevisiae*: low density, *C. reinhardtii*: high density; (III) *S. cerevisiae*: high density, *C. reinhardtii*: low density; (IV) *S. cerevisiae*: high density, *C. reinhardtii*: high density. (**b**) Values of predicted cell density divided by the measured value are plotted on the distribution diagram. The colored plots in black, red, blue, and yellow correspond to the values given by the compartments of (I), (II), (III), and (IV) in Figure 8a, respectively. (Evaluation for accuracy ratios of cell density predictions per sample using absorbance spectra data).

The accuracy of the cell density prediction was assessed for the cell-containing solutions of *S. cerevisiae* and *C. reinhardtii* by dividing each cell density into four parts (Figure 8). Regardless of the density of the cell-containing solution, few predicted values of the cell densities were higher than the measured values. When the cell density of both strains was low in the cell-containing solution, the prediction accuracy was low, varying within a range of about 0.1 to 10 for the accuracy ratio of the cell density. When the measured density of either *S. cerevisiae* or *C. reinhardtii* was high, on the low-density side, the evaluation results showed a ratio of about 30. When both strains were on the high-density side in the solution, the variation was suppressed to about 0.2 to 1.5. According to the above results, as the properties of the cell density prediction using absorbance spectra, the improvement of the prediction accuracy was confirmed when the cell-containing solution was composed of a high cell density.

In the cell-containing solution composed of *S. cerevisiae* and *C. reinhardtii*, the prediction accuracy was evaluated at each cell density. As a result, there was a tendency for the scatter to decrease for the cell density evaluation in the high-density section, indicating the possibility that the properties of the absorbance spectra of the strains could appear in the cell-containing solution. In fact, in the analytical section (IV), where both strains were at high concentrations, 87% of the evaluation accuracy of the cell density of the cell-containing solution was within the range of 0.5 to 1.2, demonstrating highly accurate analytical results.

## 4. Conclusions

In this study, a method to predict the cell densities in the mixture of *S. cerevisiae* and *C. reinhardtii* by using absorbance at each wavelength as a feature was developed. By using the prediction, the cell density in the cell-containing solution composed of *S. cerevisiae* and *C. reinhardtii* could be evaluated as *R*^2^ ˃ 0.84, depending on the absorbance spectrum. Additionally, as a result of the SHAP analyses, based on the meaningful features such as the Soret and Q bands observed in *C. reinhardtii*, the cell density prediction using absorbance spectra data could show the possibility to evaluate each cell density in the cell-containing solution of *S. cerevisiae* and *C. reinhardtii* using simple absorbance spectra data. Although the extremely randomized trees model was used as a machine learning algorithm in this study, there is the potential to improve the prediction performance by using other machine learning algorithms. Therefore, evaluating other machine learning algorithms is one of the future works. Since the data were simply the absorbance spectra of the cell-containing solution, the cell density prediction using absorbance spectra data could have an industrial impact. In the near future, based on AI analysis technology for microbiota such as in this research, meta-omics analyses using machine learning AI with data, such as transcriptomics and metabolomics, obtained from microbiota composed of multiple microorganisms, could be used to understand the metabolic flow related to the production of value-added compounds.

## Data Availability

The datasets generated and/or analyzed during the current study are available from the corresponding author on reasonable request.
